# Development of a Human Cytomegalovirus (HCMV)-Based Therapeutic Cancer Vaccine Uncovers a Previously Unsuspected Viral Block of MHC Class I Antigen Presentation

**DOI:** 10.3389/fimmu.2019.01776

**Published:** 2019-07-30

**Authors:** Mohammed O. Abdelaziz, Sophia Ossmann, Andreas M. Kaufmann, Judith Leitner, Peter Steinberger, Gerald Willimsky, Martin J. Raftery, Günther Schönrich

**Affiliations:** ^1^Institute of Virology, Charité–Universitätsmedizin Berlin, Corporate Member of Freie Universität Berlin, Humboldt-Universität zu Berlin, and Berlin Institute of Health, Berlin, Germany; ^2^Clinic for Gynecology, Charité–Universitätsmedizin Berlin, Corporate Member of Freie Universität Berlin, Humboldt-Universität zu Berlin, Berlin Institute of Health, Berlin, Germany; ^3^Division of Immune Receptors and T Cell Activation, Institute of Immunology, Medical University of Vienna, Vienna, Austria; ^4^Institute of Immunology, Charité–Universitätsmedizin Berlin, Corporate Member of Freie Universität Berlin, Humboldt-Universität zu Berlin, Berlin Institute of Health, Berlin, Germany; ^5^German Cancer Research Center, Heidelberg, Germany; ^6^German Cancer Consortium, Partner Site Berlin, Berlin, Germany

**Keywords:** human cytomegalovirus, therapeutic cancer vaccine, glioblastoma, cancer immunotherapy, viral immune evasion

## Abstract

Human cytomegalovirus (HCMV) induces a uniquely high frequency of virus-specific effector/memory CD8+ T-cells, a phenomenon termed “memory inflation”. Thus, HCMV-based vaccines are particularly interesting in order to stimulate a sustained and strong cellular immune response against cancer. Glioblastoma multiforme (GBM) is the most aggressive primary brain tumor with high lethality and inevitable relapse. The current standard treatment does not significantly improve the desperate situation underlining the urgent need to develop novel approaches. Although HCMV is highly fastidious with regard to species and cell type, GBM cell lines are susceptible to HCMV. In order to generate HCMV-based therapeutic vaccine candidates, we deleted all HCMV-encoded proteins (immunoevasins) that interfere with MHC class I presentation. The aim being to use the viral vector as an adjuvant for presentation of endogenous tumor antigens, the presentation of high levels of vector-encoded neoantigens and finally the repurposing of bystander HCMV-specific CD8+ T cells to fight the tumor. As neoantigen, we exemplarily used the E6 and E7 proteins of human papillomavirus type 16 (HPV-16) as a non-transforming fusion protein (E6/E7) that covers all relevant antigenic peptides. Surprisingly, GBM cells infected with E6/E7-expressing HCMV-vectors failed to stimulate E6-specific T cells despite high level expression of E6/E7 protein. Further experiments revealed that MHC class I presentation of E6/E7 is impaired by the HCMV-vector although it lacks all known immunoevasins. We also generated HCMV-based vectors that express E6-derived peptide fused to HCMV proteins. GBM cells infected with these vectors efficiently stimulated E6-specific T cells. Thus, fusion of antigenic sequences to HCMV proteins is required for efficient presentation via MHC class I molecules during infection. Taken together, these results provide the preclinical basis for development of HCMV-based vaccines and also reveal a novel HCMV-encoded block of MHC class I presentation.

## Introduction

Glioblastoma multiforme (GBM) is one of the most frequent and devastating brain tumors (1, 2). In fact, GBM is incurable and has a bad prognosis even after aggressive standard treatment that combines radiation, surgery and chemotherapy (3). Accordingly, there is a need to develop novel therapeutic strategies to combat this deadly disease.

Different forms of immunotherapy have been implemented or explored in a variety of human malignancies including GBM (4). Adoptive transfer of genetically modified T cells may be an option in treatment of GBM (5–8). In recent clinical trials, checkpoint inhibitors have failed to prolong the overall survival of patients with recurrent GBM (9–11). As a neoadjuvant therapy, however, PD-1 monoclonal antibody blockade improves local and systemic antitumor T cell responses (12). Therapeutic cancer vaccines stimulating tumor-reactive CD8+ T cells represent another form of immunotherapy that has also been tested in GBM patients (4, 13).

Successful tumor immunotherapy requires preexisting CD8+ T cells in the tumor microenvironment (TME) (14, 15) and genetic mutations that generate tumor neoantigens (16, 17). GBM, however, provides a “cold” TME with low numbers of infiltrating immune cells (15, 18) and scarce somatic mutations (19, 20). *In situ* vaccination with viral vectors can turn “cold” TME into “warm” through the adjuvant effect resulting from triggering multiple pattern recognition receptors (PRRs) (21–25). This inflammatory response may increase TME infiltration with immune cells. A large fraction of tumor-infiltrating immune cells are in fact memory CD8+ T lymphocytes specific for common viruses such as human cytomegalovirus (HCMV) (26–29). These cells are neither tolerized nor exhausted by continuous stimulation and can be repurposed for tumor immunosurveillance (27).

Human cytomegalovirus (HCMV) inflates memory by intermittent reactivation from latency or reinfections (30–32). In HCMV-infected humans, on average 10% of the circulating T cells with an effector-memory phenotype are in fact HCMV-specific (33, 34). Thus, HCMV-based vectors represent a very promising novel platform for therapeutic vaccination (35, 36). HCMV persists in immunocompetent individuals without causing disease (37). Intriguingly, HCMV infects GBM cells *in vitro* (38). Moreover, HCMV is detected in GBM tumor tissue but not in the surrounding normal brain tissue (39). Thus, immunotherapy may leverage HCMV-encoded tumor antigens to induce elimination of tumor cells by cytotoxic CD8+ T cells (40–42). Several strategies to achieve this goal have been explored including adoptive transfer of *in vitro*-expanded HCMV-specific T cells and vaccination with autologous dendritic cells (DCs) stimulating HCMV-specific T cells *in vivo* (39).

In this study, we designed novel HCMV-based therapeutic viral vaccines to exploit the patient's own immune system for elimination of tumor cells. We increased the immunostimulatory capacity of the HCMV-based vector by deleting important viral immune evasion genes. Moreover, we expressed a well-characterized epitope from human papillomavirus (HPV) that functions as a neo-epitope after infection of GBM cells. Finally, we tested whether genetically altered T cells specific for HCMV-encoded epitope or neo-epitope are stimulated by GBM cells infected with the HCMV-based vaccines.

## Materials and Methods

### Ethics Statement

Buffy coat preparations were purchased from German Red Cross (Dresden, Germany). Blood samples were taken with the approval of the ethics committee of the Charité–Universitätsmedizin Berlin. Written informed consent was obtained from all donors.

### Cells

The GBM cell lines U343 and LN18 were kindly provided by the Department of Neurosurgery, Charité-Universitätsmedizin Berlin, Berlin, Germany. The GBM cell line U251 was a kind gift of L. Wiebusch from the Children's Hospital, Laboratory for Molecular Biology, Charité-Universitätsmedizin Berlin, Berlin, Germany. Human embryonic lung fibroblasts (Fi301) and GBM cell lines were cultured in Eagle's minimum essential medium (EMEM) from Lonza supplemented with 1 mM sodium pyruvate, 2 mM l-alanyl-l-glutamine, non-essential amino acids, 50 μg/ml gentamicin, and 10% heat inactivated FBS (hiFBS) (HyClone). PBMCs and reporter Jurkat cell lines were cultured in RPMI 1640 medium (Gibco) supplemented with 2 mM l-glutamine, 25 mM HEPES Buffer, 50 μg/ml gentamicin, and 10% hiFBS.

### Flow Cytometry of Surface Molecules

Cells were harvested, washed and stained as previously described (43). Cell surface expression of HLA-A2 molecules was detected by using PE-conjugated anti-HLA-A2 antibody BB7.2 (BioLegend). For quantifying fluorescence of labeled cells, a FACSCalibur® (BD Biosciences) was used. Results were evaluated with the software programs CellQuestPro® (BD Biosciences) and FlowJo V10 (Tree Star, Inc).

### Viruses

HCMV strain TB40/E and the corresponding bacterial artificial chromosome TB40/E-BAC (clone 4) as well as RV-TB40-BAC_KL7_-SE-EGFP, an enhanced green fluorescent protein (EGFP)-expressing virus derived from TB40/E (44), were kindly provided by Christian Sinzger, University of Ulm, Ulm, Germany. The advantages of TB40/E are high titer growth in cell culture similar to lab strains and cell tropism resembling recent clinical isolates (45). TB40/E and the mutants derived from TB40/E-BAC were propagated in Fi301 cells. For generation of virus stocks, cells and medium were collected at various times after infection, after which cells were disrupted by three freeze-thaw cycles and cell debris was pelleted by centrifugation.

### Generation of Recombinant Viruses

As a neoantigen for expression in TB40/E-BAC derived vectors, we used human papillomavirus type 16 (HPV-16) consensus E6/E7 fusion protein (ConE6E7, GenBank accession number: FJ229356) (46). In addition, the HLA-A2-binding peptide E6_29−38_ (TIHDIILECV) derived from the E6 protein of HPV-16 (47) was fused with an AA-linker (AATIHDIILECV) to the C-terminus of HCMV IE1 (E6peptideIE1) or HCMV UL83 (E6peptideUL83). The corresponding sequences were synthesized and verified by Integrated DNA Technologies (IDT). The synthesized E6/E7 encoding sequence was digested with EcoRI and Kpn-I and cloned into the expression vectors pEF6/V5-His A and pcDNA™3.1 (+). These constructs were named pEF6E6/E7EcoRI and pcDNAE6/E7Kpn-I, respectively. Recombinant HCMV was generated using BAC technology as previously described (48). All recombinant BAC clones were confirmed by PCR and DNA-sequencing of the target area. Viruses were reconstituted from BACs by electroporation of 1 × 10^6^ Fi301 cells using program A24 of the Nucleofector II (Amaxa) and a basic Nucleofector kit (Lonza), according the manufacturer's instructions.

### Virus Titration and Growth Kinetics

Virus titers of virus stocks and multi-step growth kinetics were quantified by 50% tissue culture infectious dose (TCID50) assay on Fi301 cells. The TCID50 values were calculated using the method of Reed and Muench (49).

### Stable Transfection of U251

U251 cells were stably transfected with pcDNAE6/E7Kpn-I by electroporation as previously described (50). Transfected cells were selected by G418 for neomycin resistance and different clones were isolated and separately cultured for E6 and E7 expression assays.

### Detection of HPV-16 E7 Protein

For detection of E6/E7 fusion protein, 1 × 10^6^ cells were trypsinized and aliquots covering a range of different cell numbers were prepared (7 × 10^2^ to 16 × 10^4^ cells). In these aliquots, the E6/E7 fusion protein was detected by using recomWell HPV 16/18/45 ELISA Kit (Mikrogen GmbH, Neuried, Germany) according to manufacturer's instructions. The optical density was measured at 450 nm in a microplate photometer (Multiskan FC, Thermo Fisher Scientific, USA). The absorbance detected for experimental probes was expressed relative to the absorbance measured for the same number of CaSki cells, an E6- and E7-expressing cervical carcinoma cells that served as positive control.

### Generation of TCR Expression Vectors

For HLA-A2-restricted HPV E6_29−36_-specific TCR (51) transgene cassettes were codon-optimized for human expression and synthesized by GeneArt/Life Technologies. TCR-α/β chains with human TCR constant regions replaced by their murine counterparts were linked via 2A “self-cleaving” peptide sequence from *Porcine teschovirus-1* (P2A) and cloned in the configuration TCRβ-P2A-TCRα into pMP71-PRE using *Not*I and *Eco*RI restriction sites as described recently (52). The HCMV-specific TCR (NLV3) detecting a HLA-A2-restricted epitope derived from pp65 (NLVPMVATV; aa 495-503) was used in its original configuration as described by Schub et al. (53).

### TCR Gene Transfer

TCR gene transfer was carried out as described (54) with minor modifications. In brief, HEK-293 cells stably expressing GALV-env and MLV-gag/pol were grown to ~80% confluence and transfected with 3 μg of pMP71-TCR vectors in the presence of 10 μg Lipofectamine2000 (Life Technologies). At 48 and 72 h after transfection, 3 ml of retrovirus containing supernatant were harvested. 1 × 10^6^ human PBMCs, that had been frozen after isolation from healthy donors by ficoll gradient centrifugation, were thawed and stimulated with 5 μg/ml anti-CD3 (OKT3) and 1 μg/ml anti-CD28 (CD28.2) (Biolegend) coated plates in the presence of 300 U/ml recombinant human interleukin 2 (hIL-2) (Peprotech). Transductions at 48 and 72 h after stimulation were performed by addition of retrovirus containing supernatant and 4 μg/ml protamine sulfate followed by spinoculation for 90 min at 800 g and 32°C (1st transduction). For second transduction, retrovirus was preloaded onto retronectin (Takara)-coated plates followed by spinoculation for 30 min at 800 g and 32°C. Transduced PBMCs were maintained in the presence of 300 U/ml hIL-2 for a total of 2 weeks. At least 2 days prior to use in experiments, transduced PBMCs were cultured in the presence of 30 U/ml hIL-2.

### Functional Assays With TCR-Transduced T Cells

IFN-γ production was measured by ELISA after 16 h coculture of 1 × 10^5^ TCR-transduced T cells with 1 × 10^5^ target cells (HCMV-vector infected or HCMV-vector infected and pulsed with the corresponding peptide). As a negative control, 1 × 10^5^ TCR-transduced T cells were cocultured with 1 × 10^5^ target cells that had been left uninfected. Stimulation with phorbol myristate acetate and ionomycin (P+I) was used as a positive control.

### Reporter Cell Lines

For detection of NFAT activation, a previously described cellular platform for analysis of TCRs was used (55, 56). In the human T cell lymphoma cell line Jurkat 76 (J76), the response elements of transcription factor nuclear factor of activated T-cells (NFAT) drive the expression of EGFP (55). The J76 cell line is a subline of cell line Jurkat E6.1 (JE6.1), which lacks expression of the TCR alpha and beta chains (57). The J76 cell line was transduced with a retroviral vector encoding HLA-A2-restricted HPV E6_29−36_-specific TCR (51). Moreover, J76 cells were co-transduced to express a HLA-A2-restricted HCMV pp65-specific TCR (NLVPMVATV; aa 495-503) and CD8 (56).

For measuring of nuclear factor 'kappa-light-chain-enhancer' of activated B-cells (NF-κB) activation a single T cell reporter cell line was used, in which the responsive element for NF-κB controls EGFP expression (58). This single reporter cell line was transduced with retroviral vector encoding HLA-A2-restricted HPV E6_29−36_-specific TCR (51) or with retroviral vector encoding the HCMV-specific TCR (NLV3), which recognizes a HLA-A2-restricted epitope derived from pp65 (NLVPMVATV; aa 495-503) (53).

### Antigen Presentation Assays Using Reporter Cell Lines

For stimulation of reporter cell lines 5 × 10^4^ GBM cells (LN18, U343, or U251 cells) were infected with HCMV-based vaccines (MOI of 5). After 2 days and 4 days, respectively, infected cells were co-cultured with HPV E6-specific reporter cells and HCMV pp65-specific reporter cells, respectively, for 24 h at a ratio 2:1. Subsequently, EGFP expression of reporter cells was determined by FACS analysis.

U251 cells stably transfected with pcDNAE6/E7Kpn-I (U251-E6/E7 cells) were used to assess the impact of HCMV infection on MHC class I presentation of the E6/E7 fusion protein. For this purpose, U251 cells were left uninfected or infected with RVTB40ΔUS11 for 3–24 h at different MOIs. RVTB40ΔUS11 lacks all known HCMV-encoded immunoevasins (US2, US3, US6, and US11) that target MHC class I presentation and does not downregulate MHC class I molecules. On uninfected and infected U251-E6/E7 cells, the existing peptide-MHC class I complexes on U251 cells were removed by acid wash as previously described (59). Briefly, 1 × 10^6^ cells were harvested, washed with PBS and subsequently washed with ice-cold citric acid buffer (pH 3) for 2–3 min. Afterwards, stripped U251-E6/E7 cells were pelleted, washed twice with EMEM, resuspended in RPMI 1640 medium and subsequently co-cultured for 18 h with the HPV E6_29−36_-specific reporter cell line, in which the responsive element for NF-κB controls EGFP expression (58). Finally, EGFP expression of reporter cells was determined by FACS analysis. In parallel, the maximal peptide stimulation was always determined by pulsing a cell aliquot with the E6 peptide (1 μg/ml) during coculture with the E6-specific reporter cell line.

### Peptide Synthesis

The peptides used for pulsing antigen-presenting cells (1 μg/ml) were synthesized by peptides & elephants GmbH (Hennigsdorf, Germany).

### Statistical Analysis

Statistical significance was determined by one-way ANOVA analysis or unpaired *t*-test. *P* values below 0.05 (95% confidence) were considered to be significant. Prism 6 software (GraphPad) was used for statistical analysis.

## Results

### Susceptibility of GBM Cells to HCMV Infection

**Figure 1:**
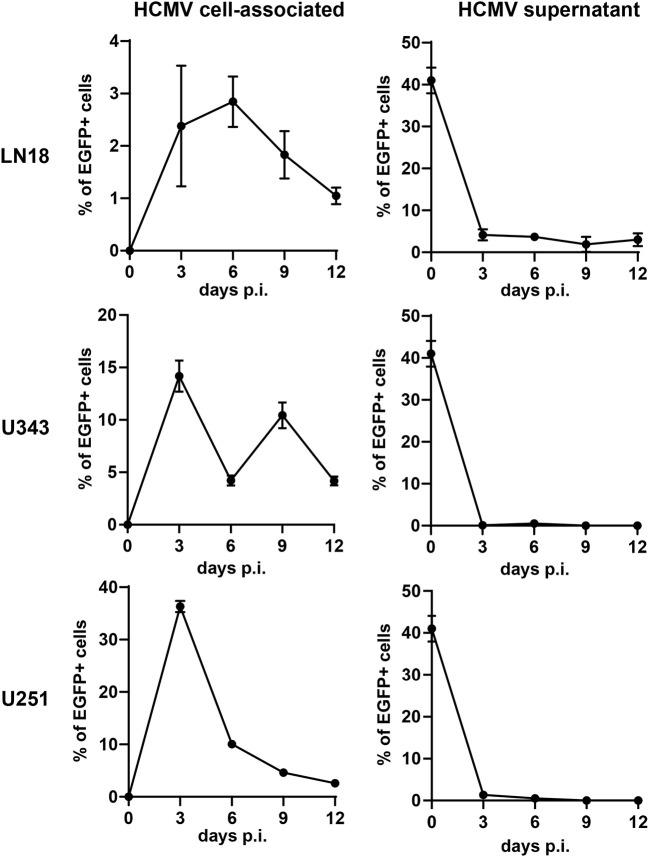
Susceptibility of GBM cells to HCMV infection. The GBM cell lines LN18, U343, and U251 were infected with RV-TB40-BAC_KL7_-SE-EGFP (MOI of 0.3). At different time points cells were tested for cell-associated virus by FACS analysis of EGFP expression (Left graphs). For detection of cell-free HCMV (Right graphs) supernatants from infected GBM cell lines were collected at different time points. Subsequently, Fi301 cells were infected with the supernatants and tested for EGFP expression by FACS 2 days after infection. Results are derived from three technical replicates; error bars represent the mean ± SEM.

In order to construct therapeutic vaccines targeting GBM we first investigated whether GBM cells are susceptible to HCMV infection. For this purpose, we used RV-TB40-BAC_KL7_-SE-EGFP. This EGFP-expressing virus is derived from low-passage HCMV strain TB40/E and contains an intact US-gene region encoding all immunoevasins (US2, US3, US6, and US11) that downregulate MHC class I presentation (44). We infected the GBM cell lines LN18, U343, and U251 with RV-TB40-BAC_KL7_-SE-EGFP at a multiplicity of infection (MOI) of 0.3. At different time points of infection, we determined the percentage of EGFP-expressing GBM cells (Figure 1, left graphs). In addition, we analyzed the presence of virus in the supernatant of infected GBM cell cultures (Figure 1, right graphs). Although all GBM cell lines tested were susceptible to HCMV, infection the virus remained mostly cell-associated during the observation period of 12 days. Thus, LN18, U343, and U251 cells are susceptible to HCMV infection as previously reported for other GBM cell lines (38, 60). Taken together, these experiments indicate that HCMV-based vectors can be used to mark GBM cells for attack by CD8+ T cells.

### Construction of HCMV-Based Therapeutic Vaccines

**Figure 2:**
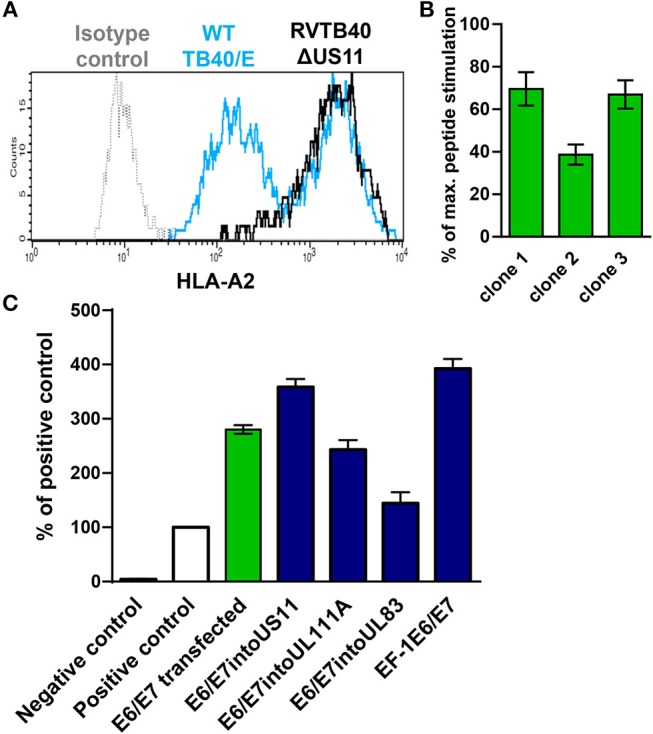
Rationale for generation of HCMV-based vectors expressing E6/E7 fusion protein. **(A)** Prevention of virus-induced MHC class I downregulation in cells infected with HCMV-based vectors lacking *US2, US3, US6*, and *US11*. Fi301 cells were infected with WT TB40/E or RVTB40ΔUS11 at MOI of 0.5. After 2 days, cells were stained with HLA-A2-specific antibody or isotype control and analyzed by flow cytometry. The results shown are representative of three experiments. **(B)** Stimulation of specific reporter T cells by U251 cells expressing E6/E7. Clones of U251 cells stably transfected with E6/E7 expressing plasmid were incubated with E6 peptide-specific reporter cell line, in which EGFP expression is driven by the responsive elements of NF-κB. Stimulation of reporter cells is given as percentage of maximal peptide stimulation, i.e., stimulation of reporter cells incubated with peptide pulsed cells. **(C)** Detection of E6/E7 in cells infected with HCMV-based vectors driving E6/E7 expression under control of endogenous or exogenous promotors. For quantification of the E6/E7 fusion protein expressed by different HCMV-based vectors (blue bars) an ELISA detecting the HPV-16 E7 protein was used. For each experimental group 6 × 10^4^ cells were used. RVTB40ΔUS11 served as a negative control. The absorbances detected for experimental probes were expressed relative to the absorbance measured for 6 × 10^4^ CaSki cells, a well-characterized E6- and E7-expressing cervical carcinoma cell line (Positive control, white bars). We also included U251 cells stably transfected with E6/E7 encoding plasmid in our analysis (green bar).

Next, we generated HCMV-based vectors that lack immunoevasins (US2, US3, US6, and US11) and efficiently stimulate CD8+ T cells. We used a bacterial artificial chromosome (BAC) clone of the HCMV strain TB40/E (TB40-BAC4), which lacks the *US1-US6* region due to insertion of the BAC (45). We obtained RVTB40ΔUS11 from TB40-BAC4 by deleting *US11*. RVTB40ΔUS11 does not downregulate MHC class I molecules as recently described (Figure 2A) (61).

**Figure 3:**
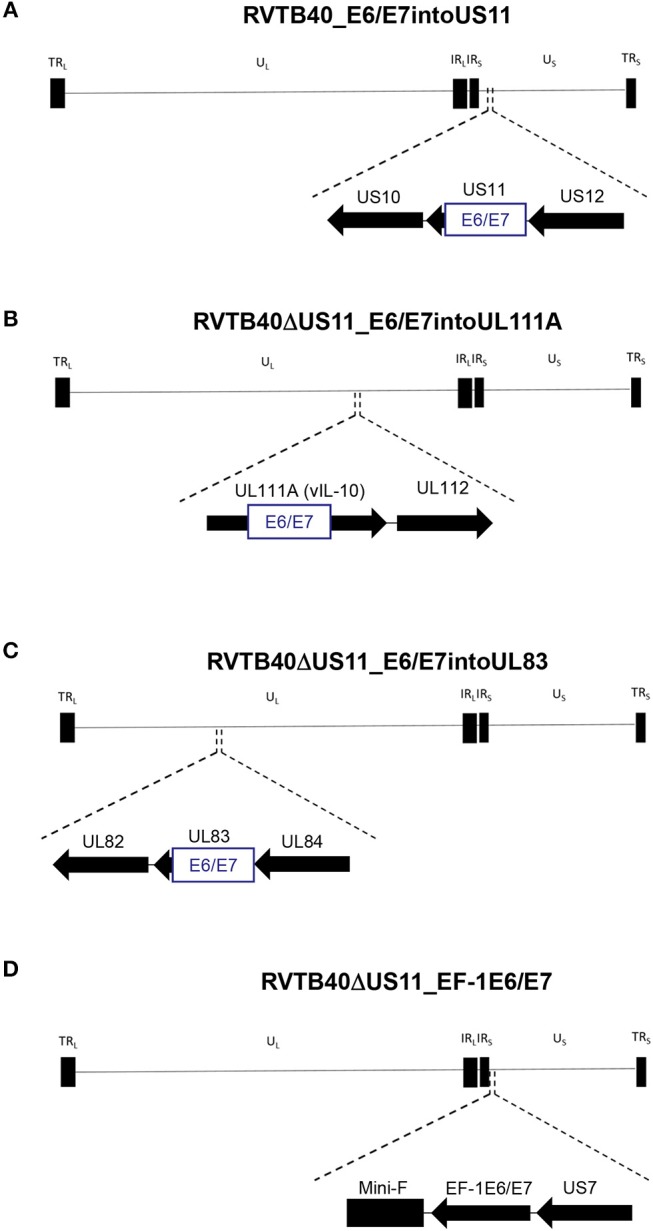
Construction of HCMV-based vaccines expressing E6/E7 fusion protein. The HCMV genome has a length of ~235 kB and contains a unique long (U_L_) and a unique short (U_S_) region each flanked by terminal (TR_L_ and TR_S_), and internal (IR_L_ and IR_S_) inverted repeats. The E6/E7 encoding sequence was inserted into **(A)** US11 (RVTB40_E6/E7into US11), **(B)** UL111A (RVTB40ΔUS11_E6/E7intoUL111A), or **(C)** UL83 (RVTB40ΔUS11_E6/E7intoUL83) in such a way that endogenous promotors control E6/E7 expression. **(D)** The E6/E7 consensus sequence was put under the control of the elongation factor-1 alpha (EF-1 alpha) promotor, a strong constitutive promotor of human origin, and inserted between TB40-BAC4 Mini-F sequence and US7 (RVTB40ΔUS11_EF-1E6/E7).

We now pursued two strategies to equip RVTB40ΔUS11 with neo-epitopes. Firstly, we used a consensus sequence encoding the E6 and E7 protein of human papillomavirus type 16 (HPV-16) as a fusion protein (E6/E7). E6/E7 covers all relevant antigenic peptides but is non-transforming (46). Vaccination of mice with a plasmid encoding E6/E7 induces a strong CD8+ T cell response and prevents growth of E6/E7 tumors (46). In accordance, we observed that E6/E7-expressing clones derived from stably transfected U251 cells (U251 cells) stimulate reporter T cells that recognize a HLA-A2-restricted peptide (E6_29−38_: TIHDIILECV) (47) (Figure 2B). Thus, we inserted the E6/E7 sequence into the RVTB40ΔUS11 at different locations ensuring that endogenous or exogenous promotors control E6/E7 expression (Figure 3). The E6/E7 expression level in cells infected with E6/E7-expressing HCMV-based vaccines was in the same order of magnitude as observed for U251 cell transfected with an E6/E7-expressing plasmid (Figure 2C).

**Figure 4:**
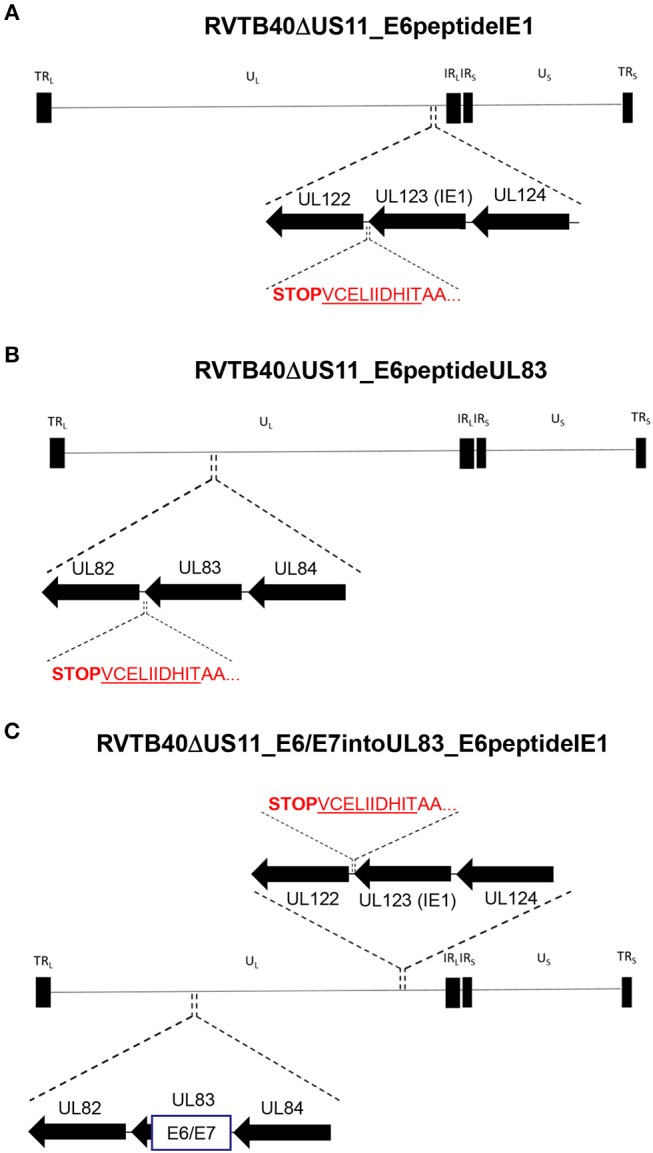
Construction of HCMV-based vaccines expressing E6 peptide fused to the C-terminus of HCMV IE1 or HCMV UL83. The HLA-A2-binding peptide E6_29−38_ (TIHDIILECV) derived from the E6 protein of HPV-16 was fused with an AA-linker (AATIHDIILECV) to the C-terminus of **(A)** HCMV UL123 (IE1) (RVTB40ΔUS11_E6peptideIE1) or **(B)** HCMV UL83 (RVTB40ΔUS11_E6peptideUL83). **(C)** In addition, a recombinant virus expressing both the E6/E7 fusion protein inserted into UL83 and the E6-peptide fused to the C-terminus of HCMV IE1 was generated (RVTB40ΔUS11_ E6/E7intoUL83_E6peptideUL83).

**Figure 5:**
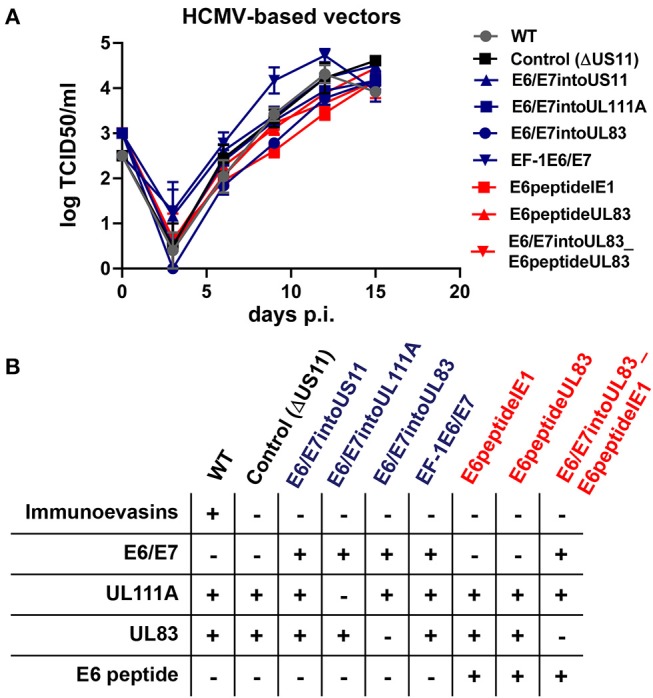
Features of HCMV-based therapeutic vaccines used in this study. **(A)** Growth curve kinetics of E6/E7 expressing vaccines (blue) and E6 peptide-expressing vaccines (red). Fi301 cells were infected at MOI of 0.01. Supernatant was collected at different time points after infection and titrated on Fi301 cells to calculate the TCID50. WT TB40/E and RVTB40ΔUS11 served as a control. Results are derived from three experiments; error bars represent the mean ± SEM. **(B)** Summary of all HCMV-based vectors used in this study.

Secondly, we fused a single neo-epitope flanked by an Alanine spacer to the C-terminus of a viral protein as recently reported for murine cytomegalovirus (MCMV) (62, 63) (Figure 4). We used HPV-16 E6_29−38_ as CD8+ T cells specific for this peptide recognize and kill HLA-A2+ tumor cells expressing E6 despite tumor-associated immune evasion mechanisms (64). This E6 peptide was fused to the C-Terminus of IE1 (RVTB40ΔUS11_E6peptideIE1) or UL83 (RVTB40ΔUS11_E6peptideUL83). We also generated a mutant virus with both the full E6/E7 sequence inserted into UL83 and the E6 peptide linked to IE1 (RVTB40ΔUS11_E6/E7intoUL83_E6peptideIE1). All generated HCMV-based vaccines showed growth kinetics similar to WT TB40/E and control virus (RVTB40ΔUS11) (Figure 5A). The relevant features of the different HCMV-based vaccines are summarized in Figure 5B.

### HCMV-Based Vaccines Expressing E6 Peptide Fused to Viral Protein but Not E6/E7 Expressing HCMV-Based Vaccines Stimulate E6-Specific T Cells

**Figure 6:**
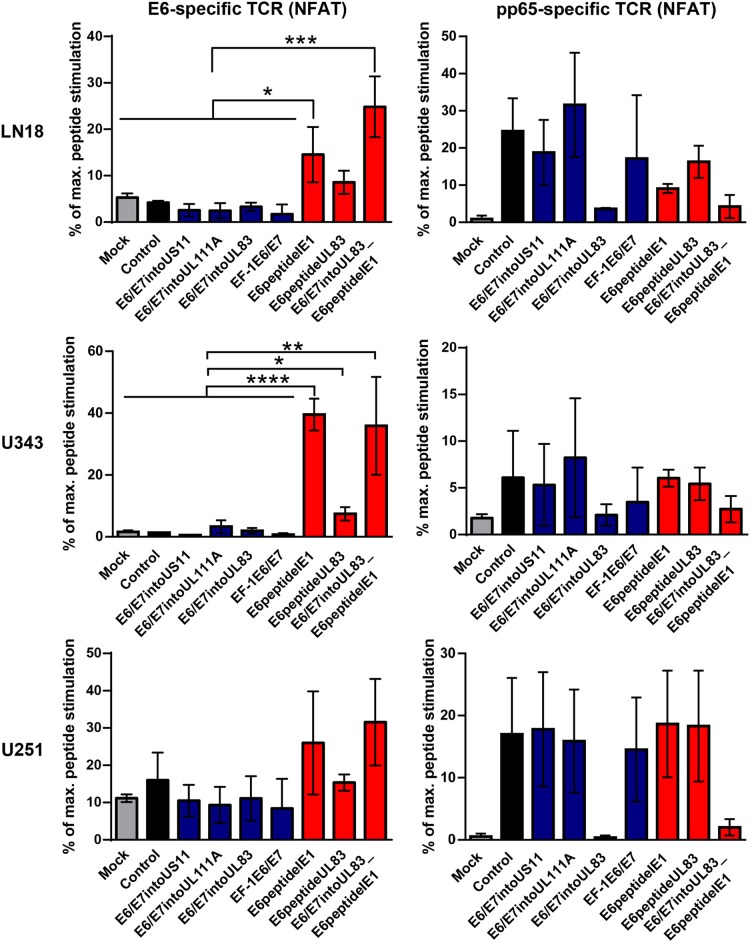
NFAT-driven EGFP expression in reporter cell lines stimulated by infected GBM cells. 5 × 10^4^ GBM cells (LN18, U343, or U251 cells) were infected with HCMV-based vaccines (MOI of 5). After 2 and 4 days, respectively, infected cells were co-cultured with HPV E6-specific reporter cells (left graphs) and HCMV pp65-specific reporter cells (right graphs), respectively, for 24 h at a ratio 2:1. Subsequently, EGFP expression of reporter cells was determined by FACS analysis. Uninfected cells (Mock) and cells infected with RVTB40ΔUS11 (Control) were also included in this type of analysis. Stimulation of reporter cells is given as percentage of maximal peptide stimulation, i.e., stimulation of reporter cells incubated with peptide pulsed cells. Results are derived from three technical replicates; error bars represent the mean ± SEM. ^****^P < 0.0001; ^***^P < 0.001; ^**^P < 0.01; ^*^P < 0.05, one-way ANOVA test.

**Figure 7:**
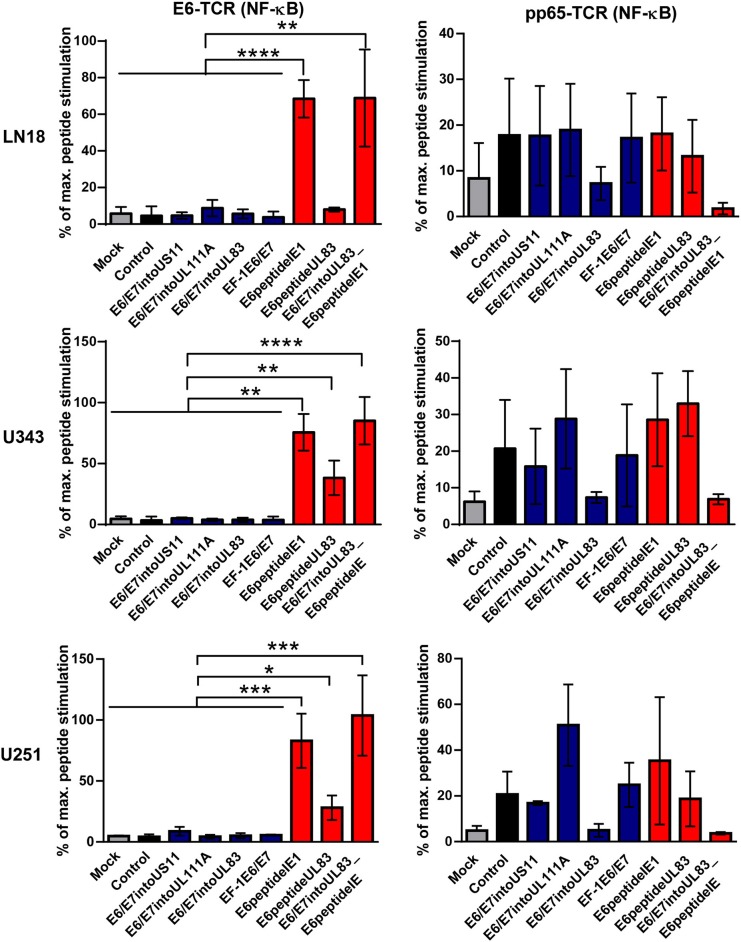
NF-κB-driven EGFP expression in reporter cell lines stimulated by infected GBM cells. 5 × 10^4^ GBM cells (LN18, U343, or U251 cells) were infected with HCMV-based vaccines (MOI of 5). After 2 and 4 days, respectively, infected cells were co-cultured with HPV E6-specific reporter cells (left graphs) and HCMV pp65-specific reporter cells (right graphs), respectively, for 24 h at a ratio 2:1. Subsequently, EGFP expression of reporter cells was determined by FACS analysis. Uninfected cells (Mock) and cells infected with RVTB40ΔUS11 (Control) were also included in this type of analysis. Stimulation of reporter cells is given as percentage of maximal peptide stimulation, i.e., stimulation of reporter cells incubated with peptide pulsed cells. Results are derived from three technical replicates; error bars represent the mean ± SEM. ^****^P < 0.0001; ^***^P < 0.001; ^**^P < 0.01; ^*^P < 0.05, one-way ANOVA test.

Now we investigated whether the different HCMV-based therapeutic vaccines could stimulate antigen-specific T cells after infection of GBM cells. To this end, we used a recently developed T cell reporter platform, in which the response elements for NFAT control EGFP expression (55, 56). These cells were transduced either with a retroviral vector encoding a HPV-specific TCR recognizing the HLA-A2-restricted peptide HPV E6_29−36_ (51) or with a retroviral vector encoding HCMV-specific TCR detecting the HLA-A2-restricted HCMV epitope pp65_495−503_ (53) together with CD8. In addition, we used another set of reporter cell lines with the same TCR specificities, in which EGFP expression is driven by the responsive elements of NF-κB (65). These reporter cell lines were incubated with HLA-A2+ LN18, U343, and U251 cells that had been infected with the different HCMV-based therapeutic vaccines for 2 or 4 days, respectively. Surprisingly, GBM cells infected with E6/E7-expressing vectors stimulated neither NFAT (Figure 6, left side, blue columns) nor NF-κB (Figure 7, left side, blue columns) in E6-specific reporter T cell lines. In stark contrast, all GBM cells infected with an HCMV-based vector expressing the E6 peptide fused with an Alanine-linker to the C-terminus of HCMV IE1 (E6peptideIE1) nicely activated NFAT (Figure 6, left side, red columns) and NF-κB (Figure 7, left side, red columns) in E6-specific T cells. Although to a lesser extent stimulation of reporter cell lines was also observed with all GBM cells that had been infected with a HCMV-based vector expressing the E6 peptide fused with an Alanine-linker to the C-terminus of HCMV UL83 (Figures 6 and 7, left side, red columns). As expected, all HCMV-based therapeutic vaccines with the exception of those deficient of pp65 (UL83) could stimulate pp65-specific reporter cell lines to a similar extent after infection of GBM cells (Figures 6 and 7, right side). Taken together, E6 peptide fused to the C-terminus of HCMV proteins but not the complete E6/E7 fusion protein expressed separately from HCMV proteins stimulated E6 peptide-specific T cells.

### A Novel HCMV-Encoded Block of MHC Class I Presentation

**Figure 8:**
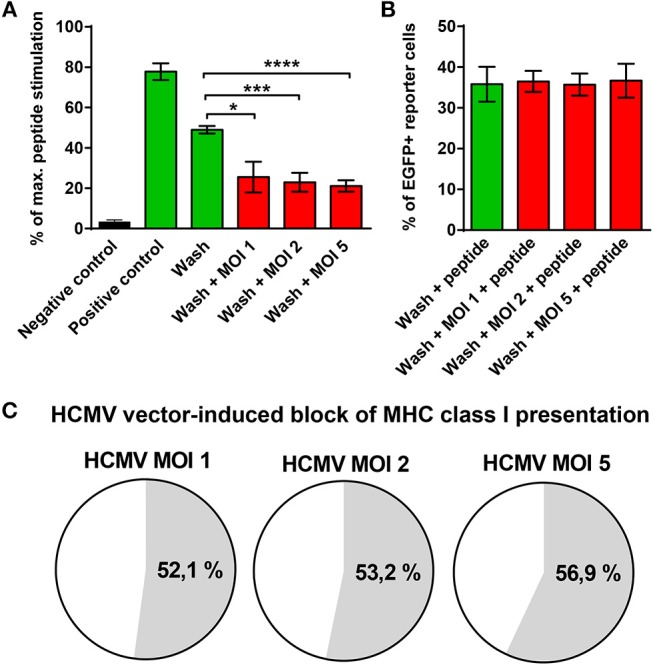
Block of MHC class I presentation induced by immunoevasin-deficient HCMV. U251 cells stably expressing the E6/E7 fusion protein (U251-E6/E7 cells) were left uninfected or infected with RVTB40ΔUS11, a mutant HCMV lacking all known immunoevasins, at the indicated MOIs for 3–24 h. Subsequently, cells were harvested, washed with ice-cold citric acid, to remove all preexisting peptide-MCH complexes and cocultured at a ratio of 2:1 with HPV E6-specific reporter cells, in which NF-κB drives EGFP. After 18 h EGFP expression was assessed by FACS analysis. Unwashed U251 cells (Negative control) and unwashed U251-E6/E7 cells (Positive control) were also cocultured with HPV E6-specific reporter cells. In parallel, maximal peptide stimulation was determined for each experimental group by pulsing cells additionally with E6 peptide (1 μg/ml) before coculture with HPV E6-specific reporter cells and subsequent FACs analysis. **(A)** The stimulation in each experimental group is given as percentage of maximal peptide stimulation. **(B)** The % of EGFP+ reporter cells after pulsing with E6 peptide (maximal peptide stimulation) is shown for washed U251-E6/E7 cells left uninfected and washed U251-E6/E7 cells infected with mutant HCMV at the indicated MOIs. **(C)** The block of MHC class I presentation after infection with mutant HCMV at the indicated MOIs is given as a percentage. The results shown are derived from three independent experiments. Error bars represent the mean ± SEM (^****^P < 0.0001; ^***^P < 0.001; ^*^P < 0.05; unpaired *t*-test).

The finding that GBM cells infected with HCMV vaccines failed to stimulate E6-specific T cells despite abundant E6/E7 protein expression was surprising. It suggested that MHC class I presentation of E6/E7 is impaired by the HCMV-vector although RVTB40ΔUS11 lacks all known immunoevasins (US2, US3, US6, US11). To address this issue, aliquots of transfected U251 cells, which stably express the E6/E7 protein, were left uninfected or infected at different MOIs with the HCMV-vector. Thereafter, cells were acid washed as described previously (59) to remove all existing peptide-MHC class I complexes from the cell surface. Subsequently, cells were co-cultured for 18 h with HPV E6_29−36_-specific reporter cells, which express EGFP under the control of NF-κB responsive elements (58). Maximal peptide stimulation was assessed in parallel by pulsing cells with E6 peptide during coculture with the reporter cells. Figure 8A shows that U251-E6/E7 cells (Positive control) but not untransfected U251 cells (Negative control) stimulated E6 peptide-specific reporter cells. Strikingly, acid washed U251-E6/E7 cells that had been infected with different MOIs of the HCMV-vector showed a significantly reduced capacity to stimulate E6-specific reporter cells as compared to acid washed uninfected U251-E6/E7 cells (Figure 8A). After additional pulsing with exogenous E6 peptide, however, acid washed infected U251-E6/E7 cells stimulated E6-specific reporter cells to a similar extent as acid washed uninfected U251-E6/E7 cells (Figure 8B). In fact, the block of MHC class I antigen presentation induced by the HCMV-vector was more than 50% (Figure 8C). Taken together, we discovered a previously unsuspected HCMV-encoded block of MHC class I presentation.

### Genetically Altered Human T Cells Secrete IFN-γ in Response to E6 Peptide but Not E6/E7 Expressing HCMV-Based Vaccines

**Figure 9:**
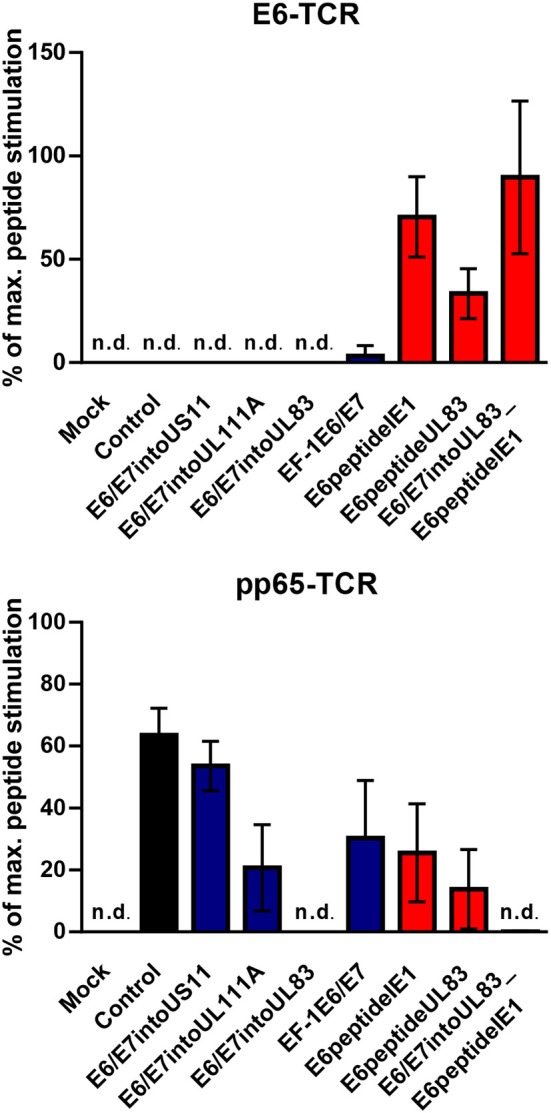
Release of IFN-γ by TCR transduced PBMCs after stimulation with infected cells. 1 × 10^5^ human PBMCs, transduced with HPV E6-specific TCR and HCMV pp65-specific TCR), respectively, or non-transduced (Background), were co-cultured for 16 h with 1 × 10^5^ fibroblasts that had been infected for 48 h with HCMV-based vaccines. PBMCs as described above were also co-cultured with infected fibroblasts that had been additionally pulsed with the corresponding peptide (maximal peptide stimulation). Subsequently, IFN-γ production was measured by ELISA. Release of IFN-γ is shown as percentage of maximal peptide stimulation after subtraction of the background. Uninfected cells (Mock) and cells infected with RVTB40ΔUS11 (Control) were also included in this type of analysis. Results are derived from three experiments; error bars represent the mean ± SEM; n.d., not detectable.

HCMV-based vaccines enabling presentation of a neo-epitope by tumor cells could be combined with adoptive transfer of genetically modified T cells specific for the vectored neo-epitope. In order to test this option and verify our results obtained with the reporter cell lines, we transduced human PBMCs with either retroviral vector encoding E6-specific TCR or retroviral vector encoding pp65-specific TCR. Subsequently, we co-cultured these cells for 16 h with vaccine-infected fibroblasts that express HLA-A2. Untransduced PBMCs were included as a negative control and treated in the same way. After co-culture the release of IFN-γ was measured as a read out of T cell function. Moreover, we pulsed aliquots of the vaccine-infected fibroblasts with the corresponding E6-derived and pp65-derived peptides, respectively. These cells were also co-cultured with transduced PBMCs to assess the maximal peptide-stimulated IFN-γ release. In Figure 9, the specific IFN-γ release induced by vaccine-infected fibroblasts is given as a percentage of IFN-γ release after stimulation with cells that had been additionally pulsed with exogenous peptide (maximal peptide stimulation). As observed for reporter cell lines, cells infected with HCMV-based vaccines expressing E6/E7 protein did not stimulate PBMCs transduced with E6 peptide-specific TCR (Figure 9, upper graph, blue columns). In contrast, cells infected with HCMV-based vaccines expressing the E6 peptide fused to the C-terminus of HCMV IE1 or HCMV UL83 induced IFN-γ release by E6-specific PBMCs (Figure 9, upper graph, red columns). Moreover, all HCMV-based vaccines with intact *UL83* (pp65) were able to activate PBMCs transduced with pp65-specific TCR (Figure 9, lower graph). Taken together, PBMCs transduced with E6-specific TCR could be used for adoptive transfer to detect tumor cells targeted by E6 peptide-expressing HCMV-based therapeutic vaccines.

## Discussion

In this study, we generated HCMV-based therapeutic vaccines that lack immunoevasins for *in situ* vaccination of GBM patients. We pursued two different strategies to channel a defined vector-encoded neo-epitope into the processing machinery of antigen-presenting cells. In one set of HCMV-based vaccines, we expressed the consensus sequence encoding an immunogenic but non-transforming E6/E7 fusion protein under the control of endogenous or exogenous promoters. In another set, we fused a single E6-epitope to the C-terminus of HCMV IE1 or HCMV UL83. Surprisingly, GBM cells transfected with an E6/E7 expression plasmid but not cells infected with the E6/E7 expressing HCMV-vectors were recognized by E6-specific T cells despite comparable E6/E7 expression. In contrast, cells infected with HCMV-based vaccines expressing an E6-epitope fused to HCMV proteins by an Alanine linker nicely stimulated E6 peptide-specific T cells. Subsequent analysis demonstrated a previously unnoticed HCMV-encoded block of MHC class I presentation that could explain the failure of E6/E7-expressing vaccines.

The central nervous system is subjected to continuous immunosurveillance through special gateways that allow exchange of immune cells and antigens with the periphery (66). As outlined in a recent review (67), antigens in the CNS are transported to cervical lymph nodes either in a soluble form or via APCs that take up antigen in the meningeal linings. After priming in the CNS-draining lymph nodes, antigen-specific T cells home back to the CNS to kill their target. Thus, *in situ* vaccination with a HCMV-based therapeutic vaccine in the brain can activate specific cytotoxic T cells in the CNS-draining lymph nodes. These in turn can migrate back to the CNS to eliminate tumor cells. We found that LN18, U343, and U251 cells are susceptible to HCMV infection as previously reported for other GBM cell lines (38, 60). Moreover, it has been recently shown that HCMV targets Glioma stem–like cells (GSCs) (60, 68). GSCs are radioresistant and chemoresistant and play a crucial role in progression and recurrence of tumor cells. Accordingly, they represent attractive targets for novel GBM therapies (69). HCMV-based therapeutic vaccines expressing E6 peptide as a neo-epitope and lacking immunoevasins could render these tumor-driving cells vulnerable to cytotoxic attack by E6-specific CD8+ T cells. After killing of GSCs release of apoptotic debris containing further tumor-specific antigens could be phagocytosed by resident microglia or brain endothelial cells, which efficiently cross prime CD8+ T cells (70, 71). In addition, many viruses including HCMV can trigger bystander activation of antiviral memory CD8+ T cells as part of an early line of antiviral defense (72–77). Thus, therapeutic HCMV-based vaccines as described in this study could amplify the anti-tumor response in GBM patients by several distinct mechanisms.

HCMV-based vaccines expressing the E6-epitope fused to the C-terminus of HCMV IE1 or HCMV UL83 could easily activate E6-specific T cells. In accordance, MCMV-vector expressing a HPV E7-derived peptide at the C-terminus of MCMV IE2 protein could efficiently protect mice from lethal tumor challenge (62, 78). In contrast, cells infected with HCMV-based vaccines expressing the E6/E7 protein separately from viral proteins did not stimulate E6-specific reporter cell lines or E6-TCR transduced PBMCs despite strong E6/E7 expression. The fusion protein E6/E7, however, was not *per se* resistant to processing. Uninfected U251 cells stably transfected with pcDNA-E6/E7 (U251-E6/E7 cells) expressed E6/E7 at the same order of magnitude and stimulated E6-TCR expressing reporter cells. Thus, the E6 epitope is naturally processed and presented by HLA-A2 in the absence of HCMV.

After infection of U251-E6/E7 cells with HCMV, however, the MHC class I presentation of the E6 peptide derived from the E6/E7 fusion protein was impaired. This was not due to known HCMV-encoded immunoevasins as we used RVTB40ΔUS11 as a vector. This mutant HCMV lacks US2, US3, US6, and US11, the known immunoevasins. It is well-described that cytosolic and nuclear proteasomes have to degrade viral proteins to generate the viral peptides that are presented by MHC class I molecules on the cell surface (79). On the other hand, viral pathogens such as herpes simplex viruses and HCMV highjack and relocalize the proteasomal machinery of the host cells to facilitate their own replication (80–82). Thus, these pathogens may diminish the proteasomal activity for processing of antigens thereby reducing the presentation of peptides by MHC class I molecules. The precise mechanism underlying this novel virus-induced block of MHC class I presentation remains to be elucidated.

We observed that GBM cells infected with HCMV-based therapeutic vaccines stimulate IFN-γ release by pp65-sepcific T cells. In fact, pp65 is the most abundant HCMV-encoded protein (83) and represents a major target for the CD8+ T cell responses in infected human individuals (84, 85). It may be a useful target for immunotherapeutic interventions in GBM patients as pp65-specific cytotoxic T cells lyse HCMV-infected GBM cell lines *in vitro* (86, 87). Thus, PBMCs derived from GBM patients could be transduced *in vitro* with retroviral vectors encoding pp65-TCR and adoptively transferred back to eliminate GBM cells. Experiments with rhesus CMV in rhesus macaques, an animal model for HCMV infection of humans, have demonstrated that pp65-specific T cell responses are important for limiting viral dissemination during primary infection (88). This result implies that simultaneous application of pp65-specifc T cells with *in situ* vaccination of HCMV-based therapeutic vaccines prevents unwanted side effects due to virus spread. Thus, although pp65 helps HCMV to subvert host defense (89–93) and is not required for viral replication (94) it should not be eliminated from a HCMV-based therapeutic vaccine. On the other hand, it is important to use HCMV-based vectors, which do not express cmvIL-10 (UL111A) for several reasons. Firstly, cmvIL-10 dampens the antiviral immune response (95–100). Secondly, cmvIL-10 produced by HCMV-infected GSCs can induce immunosuppressive macrophages and microglia, which subsequently support tumor growth (42, 101).

Autologous DC vaccines generated *ex vivo* from peripheral blood monocytes represent another promising novel approach in immunotherapy of GBM patients (40, 41, 102–104). They can complement adoptive T cell transfer and *in situ* vaccination and play a role in adjuvant treatment of cancer including GBM (105). HCMV-based vectors may be useful for generation of DC vaccines because HCMV infects DCs (43, 106). However, cmvIL-10 confers an immunosuppressive function upon HCMV-infected DCs (95–100, 107). Thus, HCMV-based vaccines lacking cmvIL-10 may be suitable for generation of autologous DCs that stimulate pp65-specific T cells and neo-epitopes expressed by the HCMV-based vector.

Besides GBM cells HCMV also infects cells from other malignant human tumors including colorectal carcinoma and prostate cancer (108–110). Accordingly, patients with these malignancies could also benefit from vaccination with HCMV-based therapeutic vaccines expressing neo-epitopes.

## Data Availability

All datasets generated for this study are included in the manuscript and/or the supplementary files.

## Author Contributions

MA designed research, performed experiments, analyzed data, and contributed to figure preparation. SO performed experiments. AK designed experiments and provided intellectual input. JL and PS contributed new reagents, analytic tools. GW contributed new reagents, analytic tools, designed experiments, analyzed data, and provided intellectual input. MR designed research, analyzed data, provided intellectual input and contributed to manuscript writing. GS was involved in experiment conception, wrote the paper, analyzed data, provided intellectual input and prepared figures.

### Conflict of Interest Statement

The authors declare that the research was conducted in the absence of any commercial or financial relationships that could be construed as a potential conflict of interest.
